# Factors Predicting Detrimental Change in Declarative Memory Among Women With HIV: A Study of Heterogeneity in Cognition

**DOI:** 10.3389/fpsyg.2020.548521

**Published:** 2020-10-15

**Authors:** Kathryn C. Fitzgerald, Pauline M. Maki, Yanxun Xu, Wei Jin, Raha Dastgheyb, Dionna W. Williams, Gayle Springer, Kathryn Anastos, Deborah Gustafson, Amanda B. Spence, Adaora A. Adimora, Drenna Waldrop, David E. Vance, Hector Bolivar, Victor G. Valcour, Leah H. Rubin

**Affiliations:** ^1^Department of Neurology, Johns Hopkins University School of Medicine, Baltimore, MD, United States; ^2^Departments of Psychiatry and Psychology, University of Illinois at Chicago, Chicago, IL, United States; ^3^Department of Applied Mathematics and Statistics, Johns Hopkins University, Baltimore, MD, United States; ^4^Division of Biostatistics and Bioinformatics, The Sidney Kimmel Comprehensive Cancer Center, Johns Hopkins University School of Medicine, Baltimore, MD, United States; ^5^Department of Molecular and Comparative Pathobiology, Johns Hopkins University School of Medicine, Baltimore, MD, United States; ^6^Division of Clinical Pharmacology, Johns Hopkins University School of Medicine, Baltimore, MD, United States; ^7^Department of Epidemiology, Johns Hopkins Bloomberg School of Public Health, Baltimore, MD, United States; ^8^Albert Einstein College of Medicine and Montefiore Medical Center, Bronx, NY, United States; ^9^Department of Neurology, SUNY Downstate Medical Center, Brooklyn, NY, United States; ^10^Division of Infectious Diseases, Georgetown University Medical Center, Washington, DC, United States; ^11^Division of Infectious Diseases, The University of North Carolina at Chapel Hill, Chapel Hill, NC, United States; ^12^Nell Hodgson Woodruff School of Nursing, Emory University, Atlanta, GA, United States; ^13^School of Nursing, University of Alabama at Birmingham, Birmingham, AL, United States; ^14^University of Miami Miller School of Medicine, Miami, FL, United States; ^15^Memory and Aging Center, Department of Neurology, University of California, San Francisco, San Francisco, CA, United States; ^16^Department of Psychiatry, Johns Hopkins University School of Medicine, Baltimore, MD, United States

**Keywords:** phenotyping, longitudinal, women, declarative memory, HIV

## Abstract

**Objective:**

Statistical techniques used to study cognitive function in HIV typically yield normative estimates and can mask the heterogeneity in cognitive trajectories over time. We applied a novel statistical approach to identify clusters of individuals with distinct patterns of change in declarative memory in HIV-seropositive (HIV+) and HIV-seronegative (HIV−) women.

**Methods:**

1731 women from the Women’s Interagency HIV Study, a multi-center, prospective cohort study, completed the Hopkins Verbal Learning Test-Revised (HLVT-R) at >2 visits. To derive subgroups with similar patterns of decline by HIV-serostatus, we used a mixed-effects framework that modeled the trajectory of multiple declarative memory outcomes over time, while simultaneously clustering individuals.

**Results:**

Of the 1731 participants, 1149 were HIV+ (70% Black/African American [AA]; 30% White/Other [W/O]) and 582 were HIV− (68% AA; 32% W/O). Race stratification was necessary to optimize clustering. Among HIV+AA’s, four subgroups emerged: a subgroup with minimal decline, two with accelerated decline, and one with stable but low performance. In HIV− AA, three subgroups emerged: one with minimal decline and two with accelerated decline. In multivariable-adjusted models among HIV+, individuals with accelerated decline were less educated (*P* < 0.001) and more likely to have a history of depression (*P* < 0.001) versus those with minimal decline. Similar subgroups were identified in W/O HIV+ and W/O HIV− participants.

**Conclusion:**

We identified clinically meaningful subgroups of women with distinct phenotypes of declarative memory decline, which depend on race and HIV-serostatus using a data driven approach. Identification of underlying mechanisms and risk factors contributing to the observed differences are warranted. More broadly our modeling approach could be other populations to identify risk factors for accelerated cognitive decline and to personalize interventions.

## Introduction

Neurocognitive impairment (NCI) remains a clinically significant problem among people with HIV (PWH) and serves as a reliable predictor of everyday functioning, including financial and medication management, driving, multitasking, and vocational functioning (Heaton et al., 1996, 2004; Waldrop-Valverde et al., 2010; Scott et al., 2011). One potential key to advancing our understanding of NCI among PWH is to acknowledge and account for the considerable heterogeneity in the degree and pattern of NCI within PWH. There is mounting evidence that NCI in PWH is better characterized by heterogeneity in the degree and pattern of NCI than by homogeneity (Brouillette et al., 2016; Rubin and Maki, 2019). Heterogeneity in cross-sectional neuropsychological profiles and longitudinal neurocognitive trajectories in PWH suggests that NCI may be comprised of multiple neurocognitive conditions with potentially mixed etiologies and/or different predictors (e.g., a different underlying clinical or genetic risk profile, for example) that may have different implications for the treatment of NCI in PWH (Brouillette et al., 2016; Gomez et al., 2019; Rubin and Maki, 2019). Thus, recognizing and unpacking heterogeneity in NCI holds promise in advancing precision medicine approaches to treatment.

Using standard approaches (e.g., mixed effects regression models) for characterizing NCI in the Women’s Interagency HIV Study (WIHS), we previously examined population-level changes occurring over a 4-year period in approximately 1000 women. We observed that overall HIV-serostatus was associated with modest decrements in neurocognitive performance (Rubin et al., 2017b). When decrements were present, they were largest in the domain of verbal learning and memory (Rubin et al., 2017b). While aggregate measures of NCI, such as these group-level differences can point to specific neurocognitive domains that warrant further study, a logical next step would be to identify characteristics of PWH who show the greatest initial deficits and rates of decline in a specific domain. Specifically, a focus on declarative memory holds considerable importance as it is one of the primary domains impacted in those with amnestic cognitive impairment, a mild cognitive impairment subtype that is associated with increased risk of the progression to Alzheimer’s-type dementia (Petersen et al., 1999; Petersen, 2004).

Here, we applied a novel statistical method to identify clusters of individuals with distinct patterns of age-related change in declarative memory in HIV-seropositive (HIV+) and HIV-seronegative (HIV−) women. Our modeling approach allows us to simultaneously assess multiple declarative memory test measures and permits potential individual-level heterogeneity in decline over time. The goal is to identify HIV+ women with the most rapid declarative memory decline and to determine what factors differentiate those individuals from others. The identification of such subgroups is a critical first step in the development of ideal targeted interventions with the best chance for success, as this approach allows categorization in specific groups with similar trajectories and risk factors for declarative memory decline. Although a myriad of factors are linked to severity of neurocognitive function among PWH, our work would suggest that mental health factors (e.g., depression) (Rubin et al., 2015, 2017a; Rubin and Maki, 2019) and substance use (Meyer et al., 2013) would be strong predictors of deficits or detrimental rates of change in declarative memory among HIV+ women. Importantly, we also consider analyses stratified by race in the identification of clusters of age-related change in declarative memory, which allows us to identify whether predictors of declarative memory change varies within racial groups. Race stratification is important given that key social determinants linked to race in the United States (e.g., education) may be important factors driving these differences.

## Materials and Methods

### Study Population

All participants were enrolled in the Women’s Interagency HIV Study; full details of the design and ongoing follow-up are described in detail at http://wihshealth.org. Briefly, the first three waves of enrollment occurred between October 1994 and November 1995, October 2001 and September 2002, and January 2011 and January 2013 from Brooklyn, Bronx, Chicago, Washington DC, Los Angeles, and San Francisco. A more recent wave of enrollment occurred at sites in the southern US (Chapel Hill, Atlanta, Miami, Birmingham, and Jackson) between October 2013 and September 2015 (Adimora et al., 2018). In total 2058 HIV+ and 568 demographically matched HIV− women aged 16–74 were enrolled. Barkan et al. (1998) and Bacon et al. (2005) provide comprehensive descriptions of recruitment protocols and eligibility criteria. At semi-annual WIHS study visits, participants provide demographic (age, race, ethnicity, years of education, household income, employment status), lifestyle (smoking status, alcohol use, illicit drug use in the prior 6-months), and clinical information including information on combination antiretroviral drug therapy (cART) usage and class, depressive symptomatology (via Center for Epidemiological Studies Depression Scale; CES-D), body mass index (BMI), systolic and diastolic blood pressure, and diabetes. At each visit, participants also provide blood samples for assessment of CD4 count, plasma HIV RNA level and hepatitis C (HepC) status. Viral suppression (VS) was defined as plasma HIV RNA below limits of detection for specific assay used. WIHS was approved by the institutional review board at each site and was compliant with the Health Insurance Portability and Accountability Act. All participants provided written consent.

### Neuropsychological Tests

Beginning in 2009 and occurring approximately bi-annually thereafter, 1752 participants completed at least two batteries of neuropsychological tests including an assessment of declarative memory from the Hopkins Verbal Learning Test-Revised (HVLT-R). Women completed a mean 3.2 ± 0.89 neuropsychological assessments (Number of women with 2 assessments: 549 women; with 3: 336 women; with 4+: 846 women; Supplementary Table 1). WIHS cognitive assessments employ parallel versions of the HVLT-R, and participants have not completed repeated assessments of the same form. We included the following declarative memory measures in our analyses: single trial learning (total words recalled on Trial 1), learning (total learning), memory (delay free recall), and recognition (number of words correctly identified on a yes/no recognition test). In addition, we evaluated if the derived clusters of individuals with similar patterns of declarative memory changes also exhibit similar patterns of decline on other cognitive domains in order to evaluate the sensitivity and specificity of our findings in relation to declarative memory (e.g., do those with rapidly declining declarative memory trajectories also exhibit rapidly declining motor function?). For this analysis, we selected motor function (time to completion of grooved pegboard test for dominant and non-dominant hands) as the additional domain; we have previously shown that motor function is likely to be impaired in PWH and that motor function may decline more rapidly in this population relative those without HIV (Rubin et al., 2017b). As previously described in detail elsewhere, we transformed responses for declarative memory tests to linear scaled scores for each outcome so that test scores were standardized to a population mean (M) of 10 (among the HIV− women in WIHS) and standard deviation (SD) of 3 (Cysique et al., 2009, 2011). Scores were inspected for normality and for distributional similarity for both HIV− and HIV+ women.

### Statistical Analysis

We adopted a statistical approach that would yield subgroups of participants with similar patterns of decline in declarative memory and that would account for multiple declarative memory outcomes. To derive clusters, we adapted a novel modeling strategy originally developed for time-series gene expression data that simultaneously considers multiple longitudinal declarative memory outcomes (Sun et al., 2017). Relative to traditional longitudinal trajectory models that consider a single outcome at time (e.g., a model which estimates the rate of change in verbal learning over time and a separate model which estimates the rate of change in delay free recall over time), this analytic method considered a set of longitudinal declarative memory measures simultaneously while also accounting for correlation among those measures within and between participants. Thus, rather than identifying clustering groups for each individual measure (e.g., after applying traditional clustering methods for four declarative memory outcomes, four separate sets of clusters would be identified) which may be challenging to interpret, a single clustering set was derived that reflected grouping across the four longitudinal declarative memory outcomes (e.g., different component tests of the HVLT). Briefly, the strategy employed a Dirichlet process mixture model that adopted a linear mixed-effects framework to model the trajectory of declarative memory measures over time, and that simultaneously conducted a clustering procedure based on the regression coefficients obtained for each individual measure. To account for the correlations among memory measures, we applied a factor analysis for regression coefficients and adopted a Dirichlet-process prior distribution in the calculation of the means of regression coefficients to induce clustering. For this analysis, both intercepts and slopes were used for clustering. We also fit 20,000 iterations (10,000 were burned) using the *BClustLonG* function in the “BClustLonG” package in R using default values for hyperparameters. The posterior similarity matrix (to be used for final clustering) was calculated using the cluster membership indicator for each iteration (using the *calSim* function). We then calculated the optimal number of clusters using clustering that maximizes the posterior expected Rand adjusted index (PEAR) using the average maximinzation method (using the *maxpear* function). Sensitivity analyses compared automatic clustering detection methods with used hierarchical clustering of the posterior similarity matrix using pre-specified numbers of clusters ranging from 3 to 6 and using visual inspection of similarity matrix to confirm the optimal number of clusters. To reduce potential undue influence of small clusters of women (as these women may be outliers), we included only clusters including at least 15 individuals.

We compared patterns of change in derived clusters across individual declarative memory tests. We also evaluated how patterns of changed in the identified clusters for declarative memory compared to patterns of change in other neuropsychological tests correlated with motor domains. We then assessed whether demographic, lifestyle, or clinical characteristics were associated with membership in each trajectory group of declarative memory change using generalized linear models adjusted for age, ethnicity, and years of education. We classified each exposure group in two ways (1) a baseline exposure level (at first neuropsychological testing visit) and (2) a cumulative exposure level defined as the percentage of visits prior to neuropsychological testing in which the individual was ‘exposed’ (e.g., if an individual reports smoking at 10 of 30 visits prior to neuropsychological testing, the cumulative smoking ‘exposure’ level is calculated as 0.33). The cumulative exposure variable takes advantage of the long-term longitudinal information on exposures available in WIHS, which we hypothesized could also influence declarative memory trajectory. Cumulative exposure variables were calculated for the following variables: low income (<$12,000 per year), employment, depression, heavy drinking, marijuana use, crack/cocaine use, heroin use, CD4 counts < 200, HIV RNA ≤ 48 cp/mL, and HIV RNA ≥ 10,000 cp/mL. We use age as the time scale and for participants with missing data on declarative memory outcomes we applied a single imputation approach (using the mean when participants were missing one of the declarative memory tests). Missingness on individual declarative memory tests was relatively rare; approximately 99% of included visits were complete. We also performed sensitivity analyses where we restricted the population to virally suppressed HIV+ individuals (HIV+VS) at all visits throughout follow-up. To mitigate potential differences in longitudinal cognitive trajectories that differ by HIV-serostatus or by race (Black/African American, White/Other), we employed stratified models. Stratified analyses were necessary as initial clustering resulted in clusters that were largely dependent on race (e.g., one cluster was largely Black/African American women and another was largely White/Other women). Therefore, since it would be difficult to evaluate differences between the clusters that were independent of race, we employed stratified models in all follow-up analyses. We calculated the rate of change for each declarative memory outcome in each cluster using mixed effects models and tested for significant differences in the rate of change between clusters using likelihood ratio tests. We tested for significant differences in the distribution of demographic and clinical characteristics between cluster groups also using likelihood ratio tests.

## Results

On average, WIHS women completed 25.0 ± 14.7 (M ± SD) study visits for collection of clinical and laboratory information (15.89 ± 11.13 visits before the initiation of neuropsychological testing). We included 1731 of the 1752 participants (99%) who completed at least two neuropsychological assessments; 21 participants did not fall into a distinct cluster and were excluded. On average, participants were followed for 5.89 ± 1.83 years; there were no significant differences with respect to follow-up between racial groups (*P* = 0.67) or HIV-serostatus (*P* = 0.21).

### HIV+ Black/African American Women

**FIGURE 1:**
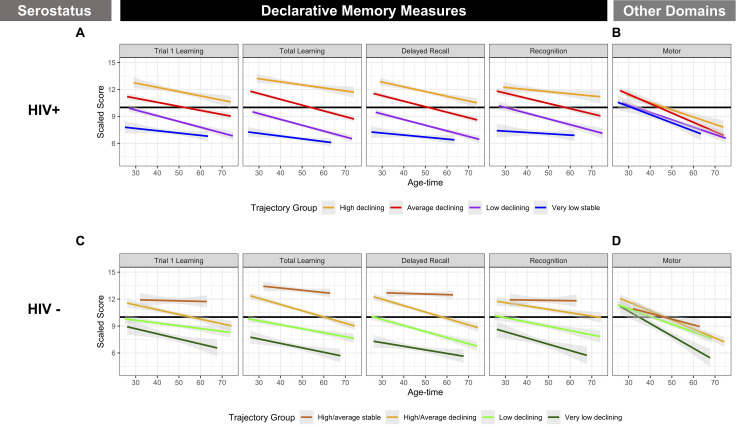
Cluster Groups in Black/African American WIHS Women. **(A)** Included declarative memory outcomes from the Hopkins Verbal Learning Test-Revised (HVLT-R): memory (delay free recall), learning (total learning), single trial learning (total words recalled on Trial 1), and recognition (number of words correctly identified on a yes/no recognition test). **(B)** Included measures of motor function (time to completion of grooved pegboard test for dominant and non-dominant hands). Values plotted are linear fit of within group averages of scaled averaged across age and time.

A summary table describing the identified clusters for each strata is provided in Supplementary Table 2. We identified four clusters of HIV-seropositive (HIV+) Black/African American women with various levels of performance at baseline and rates of decline over follow-up with relatively consistent patterns of change across declarative memory outcomes (Figure 1A). Subgroup specific patterns included a baseline high-declining group (orange; *n* = 99), a baseline average-declining group (red; *n* = 340), a baseline low-declining group (purple; *n* = 267) and a very low-stable group (blue; *n* = 95). Individuals in the high-declining group and average-declining groups had significantly faster rates of learning and memory changes relative to individuals in the very low-stable groups for total learning and recognition tests. While patterns of decline were relatively consistent across individual declarative memory tests (e.g., rates of change for declarative memory tests were similar within each cluster), similar patterns were not observed for measures of other cognitive systems, including motor function (*P* for difference in rate of change in motor function between clusters = 0.84). For example, individuals in the very low-stable declarative memory group (blue) had normal baseline scores, but rapidly declined on motor domains (Figure 1B). In multivariable models mutually adjusting for all risk factors considered, individuals in the baseline low-declining and very low-sable were significantly more likely to be less educated (*P’s* < 0.001) and unemployed (*P* = 0.005), have a history of depression (*P* < 0.001), high BMI (*P* = 0.02), and diabetes (*P* = 0.01) versus those in the high declining group; baseline age at the start of follow-up did not differ between subgroups (Table 1). We identified 3 categories of HIV+VS Black/African American women (*n* = 166; Supplementary Table 3 and Supplementary Figure 1) with varying levels of baseline performance; rates of declines between clusters were not statistically different (all *P* > 0.05). HIV+ VS Black/African American women with lower baseline performance tended to be less educated (*P* < 0.05), have lower annual household income (*P* < 0.001) and were more likely to have a history of crack/cocaine and heroin use (both *P* = 0.03) relative to those with higher baseline performance.

**TABLE 1:** Characteristics of HIV-seropositive Black/African American Women by identified cluster.

**Characteristic**	**Clustering group**				***P*-value**	
**Group [Color] in Figures 1A,B**	**High – declining [gold]**	**Average – declining [red]**	**Low – declining [purple]**	**Very low – stable [blue]**	**Unadjusted**	Multivariable adjusted*
*N*	99	340	267	95		
Average duration of time intervals between testing, mean (*M*) (Standard deviation [*SD*])	2.1 (0.4)	2.2 (0.6)	2.1 (0.5)	2.1 (0.4)		
Average duration of follow-up, years, *M* (*SD*)	4.8 (1.7)	4.7 (1.8)	4.5 (1.8)	4.5 (1.8)		
Declarative memory at baseline**						
Trial 1 learning, *M* (*SD*)	12.8 (2.2)	10.7 (2.4)	8.7 (2.4)	6.8 (2.4)	<0.0001	**<0.0001**
Total learning, *M* (*SD*)	12.8 (1.8)	10.5 (2.1)	8.4 (2.0)	6.4 (2.4)	<0.0001	**<0.0001**
Delayed recall, *M* (*SD*)	13.0 (1.8)	10.7 (2.0)	8.5 (2.0)	6.4 (2.1)	<0.0001	**<0.0001**
Recognition, *M* (*SD*)	11.9 (1.9)	10.6 (2.5)	8.6 (2.8)	6.3 (2.7)	<0.0001	**<0.0001**
*Rate of change in declarative memory per decade of age-years*						
Trial 1 learning, Beta (B) (95% CI)	−0.58 (−0.89, −0.28)	−0.61 (−0.77, −0.45)	−0.74 (−0.93, −0.56)	−0.24 (−0.60, 0.12)	0.11	0.25
Total learning, B (95% CI)	−0.40 (−0.65, −0.15)	−0.70 (−0.83, −0.57)	−0.84 (−1.00, −0.70)	−0.35 (−0.65, −0.06)	0.002	**0.01**
Delayed recall, B(95% CI)	−0.47 (−0.80, −0.13)	−0.67 (−0.84, −0.49)	−0.78 (−0.98, −0.59)	−0.43 (−0.81, −0.06)	0.24	0.26
Recognition, B (95% CI)	−0.29 (−0.62, 0.04)	−0.58 (−0.75, −0.41)	−0.85 (−1.04, −0.66)	−0.37 (−0.75, 0.02)	0.01	**0.03**
*At initial cognitive test^†^*						
Age, years, *M* (*SD*)	42.0 (7.8)	42.1 (8.7)	41.8 (8.8)	40.2 (7.5)	0.27	0.57
Hispanic ethnicity, *n* (%)	2 (2.1)	10 (3.0)	8 (3.0)	5 (5.3)	0.64	0.63
Years of education, *M* (*SD*)	14.4 (3.0)	12.8 (2.7)	11.7 (2.5)	11.1 (2.4)	<0.0001	**<0.0001**
WRAT-3 reading, *M* (*SD*)	99.6 (14.2)	93.5 (15.7)	83.4 (18.5)	80.6 (18.8)	<0.0001	**<0.0001**
Annual household income (<$12k/year), *n* (%)	30 (33.3)	140 (44.4)	153 (61.5)	62 (68.9)	<0.0001	0.11
Employed, *n* (%)	52 (54.7)	129 (38.2)	66 (25.1)	14 (14.9)	<0.0001	**0.005**
Depressed^†^, *n* (%)	17 (17.9)	91 (26.9)	85 (32.2)	49 (52.7)	<0.0001	**0.01**
Smokes, *n* (%)	32 (33.7)	137 (40.5)	118 (44.9)	50 (53.2)	0.04	0.73
Heavy drinker, *n* (%)	7 (7.5)	17 (5.0)	21 (8.0)	7 (7.5)	0.49	0.45
Marijuana use, *n* (%)	15 (16.0)	50 (14.8)	52 (19.8)	14 (14.9)	0.41	0.21
Crack, Cocaine, *n* (%)	3 (3.2)	21 (6.2)	21 (8.0)	5 (5.3)	0.36	0.53
Heroin use, *n* (%)	0 (0.0)	6 (1.8)	5 (1.9)	1 (1.1)	0.33	0.34
Body mass index, *M* (*SD*)	31.6 (8.9)	30.9 (8.5)	31.0 (8.5)	28.7 (6.9)	0.08	**0.02**
Hypertension, *n* (%)	45 (47.4)	169 (50.0)	123 (46.8)	34 (36.2)	0.13	0.81
Diabetes, *n* (%)	27 (28.4)	63 (18.6)	44 (16.7)	17 (18.1)	0.12	**0.01**
Hepatitis C RNA positive, *n* (%)	15 (15.8)	70 (20.7)	46 (17.6)	15 (16.1)	0.56	0.19
Years of ARV, *M* (*SD*)	10.0 (5.1)	8.9 (5.6)	10.1 (5.0)	9.1 (5.6)	0.01	0.63
Years of HAART, *M* (*SD*)	8.7 (4.7)	7.5 (4.9)	8.6 (4.4)	7.8 (4.8)	0.01	0.60
CD4 count < 200, *n* (%)	12 (12.6)	27 (8.0)	34 (12.9)	15 (16.13)	0.08	0.81
HIV RNA < 48 cp/mL, *n* (%)	51 (53.7)	193 (57.6)	143 (54.6)	39 (41.9)	0.06	0.26
HIV RNA > 10,000 cp/mL, *n* (%)	15 (15.8)	38 (11.3)	34 (13.0)	15 (16.1)	0.52	0.79
*% of WIHS visits prior to cognitive testing^‡^*						
Annual household income (<$12k/year), *M* (*SD*)	38.1 (35.8)	45.1 (36.0)	58.0 (34.8)	64.9 (32.6)	<0.0001	0.23
Employed, *M* (*SD*)	54.7 (36.0)	39.4 (37.3)	27.5 (33.9)	15.1 (26.9)	<0.0001	**0.0003**
Depressed, *M* (*SD*)	23.0 (26.3)	31.3 (31.1)	43.3 (34.6)	55.2 (34.7)	<0.0001	**<0.0001**
Smokes, *M* (*SD*)	40.6 (42.4)	44.6 (43.7)	47.3 (43.3)	56.9 (42.5)	<0.0001	0.20
Heavy drinker, *M* (*SD*)	5.2 (15.3)	6.9 (18.1)	8.1 (18.9)	7.9 (17.2)	0.05	0.94
Marijuana use, *M* (*SD*)	16.2 (30.7)	16.6 (29.4)	18.3 (30.2)	18.5 (28.8)	0.57	0.62
Crack, Cocaine use, *M* (*SD*)	5.0 (16.7)	10.5 (23.9)	12.5 (25.2)	9.3 (19.1)	0.86	0.07
Heroin use, *M* (*SD*)	1.1 (4.9)	2.5 (11.2)	4.0 (15.1)	4.3 (14.7)	0.16	0.32
CD4 count < 200, *M* (*SD*)	10.2 (18.2)	9.2 (18.5)	13.2 (22.4)	13.3 (24.3)	0.07	0.73
HIV RNA < 48 cp/mL, *M* (*SD*)	11.6 (18.6)	12.8 (25.5)	13.6 (25.5)	13.8 (27.7)	0.91	0.85
HIV RNA > 10,000 cp/mL, *M* (*SD*)	22.3 (25.6)	19.9 (23.8)	22.1 (23.4)	25.0 (27.0)	0.29	0.97

*^∗^Adjusted for all factors included in the table. ^∗∗^Values presented are scaled to have a mean of 10 and a standard deviation of 3. ^†^Calculated as the baseline (at initial cognitive visit) exposure level. ^‡^Calculated as a cumulative exposure level defined as the percentage of visits prior to cognitive testing in which the individual was ‘exposed’ (e.g., if an individual reports a current smoker status for 10 of 30 visits, the cumulative exposure level is defined as 0.30). M, mean; SD, standard deviation; B, beta coefficient; CI, confidence interval; HAART, highly active antiretroviral therapy; ARV, antiretroviral. Bolded p-values denote those which are < 0.05 in multivariable-adjusted models.*

### HIV− Black/African American Women

Similar to HIV+ Black/African American women, we identified four clusters of HIV− Black/African American women with various levels of performance at baseline but did not observe significant differences in the rate of change across subgroups (all *P*’s > 0.05; Figure 1C and Table 2). Subgroup specific patterns included a baseline high/average-stable group (brown; *n* = 48), a baseline high/average-declining group (orange; *n* = 150), a baseline low-declining group (light green; *n* = 148), and a very low -declining group (dark green; *n* = 51). As in HIV+ Black/African American women, while patterns of baseline performance were relatively consistent across individual declarative memory tests for each of the identified subgroups, similar patterns were not observed for changes in motor function (*P* for difference in rate of change in motor function between clusters = 0.18; Figure 1D). In multivariable models, individuals in the high/average-declining, low-declining, and very low-declining group were significantly more likely to be less educated (Table 2; *P* = 0.003), currently use crack/cocaine (*P* = 0.03), and have a history of crack/cocaine use (*P* = 0.03) versus those in the high/average-stable subgroups.

**TABLE 2:** Characteristics of HIV-seronegative Black/African American Women by identified cluster.

**Characteristic**	**Clustering Group**				***P*-value**	
**Group [Color] in Figures 1C,D**	**Black/Dashed**	**Gray/Solid**	**Gray/Dot-dash**	**Gray/Dotted**	**Unadjusted**	Multivariable adjusted*
	**High/average – stable [brown]**	**High/average – declining [golden brown]**	**Low – declining [light green]**	**Very low – declining [dark green]**		
*N*	48	150	148	51		
Average duration of time intervals between testing, years, mean (*M*) (Standard deviation [*SD*])	2.1 (0.3)	2.2 (0.5)	2.2 (0.7)	2.2 (0.5)		
Average duration of follow-up, years, *M* (*SD*)	4.3 (2.1)	4.7 (1.8)	4.6 (1.8)	4.8 (1.8)		
Declarative memory at baseline**						
Trial 1 learning, *M* (*SD*)	13.0 (2.1)	11.1 (2.4)	9.3 (2.6)	7.7 (2.7)	<0.0001	**<0.0001**
Total learning, *M* (*SD*)	13.5 (1.7)	11.6 (2.1)	9.2 (2.3)	6.4 (2.3)	<0.0001	**<0.0001**
Delayed recall, *M* (*SD*)	13.6 (1.7)	11.4 (1.9)	9.1 (2.2)	6.8 (2.3)	<0.0001	**<0.0001**
Recognition, *M* (*SD*)	11.8 (2.3)	11.1 (2.3)	8.9 (2.7)	6.7 (2.8)	<0.0001	**<0.0001**
*Rate of change in declarative memory per decade of age-years*						
Trial 1 learning, Beta (B) (95% CI)	−0.53 (−0.98, −0.08)	−0.39 (−0.60, −0.18)	−0.59 (−0.81, −0.38)	−0.56 (−1.07, −0.06)	0.61	0.48
Total learning, B (95% CI)	−0.59 (−1.06, −0.12)	−0.76 (−0.98, −0.53)	−0.80 (−1.03, −0.57)	−0.21 (−0.68, 0.27)	0.15	0.20
Delayed recall, B (95% CI)	−0.54 (−0.96, −0.12)	−0.52 (−0.72, −0.32)	−0.79 (−0.99, −0.58)	−0.50 (−0.94, −0.06)	0.20	0.11
Recognition, B (95% CI)	−0.75 (−1.21, −0.29)	−0.57 (−0.78, −0.35)	−0.37 (−0.59, −0.16)	−0.13 (−0.55, 0.48)	0.13	0.12
*At initial cognitive test^†^*						
Age, years, *M* (*SD*)	41.7 (8.6)	40.3 (10.7)	39.0 (10.8)	40.1 (8.7)	0.40	0.22
Hispanic ethnicity, *n* (%)	2 (4.3)	2 (1.4)	9 (6.3)	5 (9.8)	0.05	0.12
Years of education, *M* (*SD*)	13.6 (2.4)	13.2 (2.6)	12.1 (2.6)	10.9 (2.4)	<0.001	**0.003**
WRAT-3 reading, *M* (*SD*)	101.4 (11.7)	92.1 (15.1)	86.7 (17.4)	79.0 (18.0)	<0.001	0.15
Annual household income (<$12k/year), *n* (%)	20 (44.4)	67 (50.0)	65 (49.6)	34 (72.3)	0.02	0.73
Employed, *n* (%)	24 (47.1)	62 (43.1)	68 (47.6)	11 (23.4)	0.02	0.59
Depressed^†^, *n* (%)	9 (17.7)	35 (24.3)	45 (31.5)	22 (46.8)	0.007	0.10
Smokes, *n* (%)	25 (49.0)	68 (47.2)	70 (49.0)	25 (53.2)	0.92	0.31
Heavy drinker, *n* (%)	11 (21.6)	13 (9.0)	17 (11.9)	7 (14.9)	0.15	0.34
Marijuana use, *n* (%)	11 (21.6)	42 (29.4)	27 (18.8)	14 (29.8)	0.14	0.25
Crack, Cocaine use, *n* (%)	9 (17.7)	10 (6.9)	11 (7.7)	11 (23.4)	0.007	**0.03**
Heroin use, *n* (%)	2 (3.9)	1 (0.7)	3 (2.1)	1 (2.1)	0.50	0.10
Body mass index, *M* (*SD*)	32.9 (9.0)	32.9 (9.3)	32.4 (9.1)	31.9 (7.8)	0.89	0.77
Hypertension, *n* (%)	25 (49.0)	62 (43.1)	55 (38.5)	24 (51.1)	0.36	0.35
Diabetes, *n* (%)	12 (23.4)	29 (20.1)	25 (17.5)	11 (23.4)	0.73	0.84
Hepatitis C RNA positive, *n* (%)	4 (7.8)	17 (11.8)	14 (9.8)	5 (10.9)	0.87	0.14
*% of WIHS visits prior to cognitive testing^‡^*						
Annual household income (<$12k/year), *M* (*SD*)	34.0 (34.7)	45.1 (36.0)	52.0 (34.0)	71.3 (31.4)	<0.001	0.07
Employed, *M* (*SD*)	51.7 (38.8)	43.9 (35.7)	41.0 (33.2)	25.8 (30.4)	0.002	0.95
Depressed, *M* (*SD*)	26.1 (31.1)	28.9 (29.1)	37.5 (31.0)	53.9 (33.6)	<0.0001	0.23
Smokes, *M* (*SD*)	54.0 (44.6)	48.7 (44.4)	57.7 (43.8)	61.8 (41.1)	0.21	0.49
Heavy drinker, *M* (*SD*)	13.3 (20.6)	8.4 (19.3)	12.4 (23.4)	17.9 (25.1)	0.06	0.52
Marijuana use, *M* (*SD*)	26.5 (32.4)	22.2 (31.7)	34.3 (38.2)	30.8 (34.6)	0.03	0.07
Crack, Cocaine use, *M* (*SD*)	21.5 (33.0)	15.4 (29.3)	13.1 (24.8)	22.5 (26.5)	0.11	**0.03**
Heroin use, *M* (*SD*)	3.1 (11.2)	3.4 (13.6)	3.9 (14.6)	5.3 (15.3)	0.84	0.94

*^∗^Adjusted for all factors included in the table. ^∗∗^Values presented are scaled to have a mean of 10 and a standard deviation of 3. ^†^Calculated as the baseline (at initial cognitive visit) exposure level. ^‡^Calculated as a cumulative exposure level defined as the percentage of visits prior to cognitive testing in which the individual was ‘exposed’ (e.g., if an individual reports a current smoker status for 10 of 30 visits, the cumulative exposure level is defined as 0.30). M, mean; SD, standard deviation; B, beta coefficient; CI, confidence interval. Bolded p-values denote those which are < 0.05 in multivariable-adjusted models.*

### HIV+ White/Other Women

**FIGURE 2:**
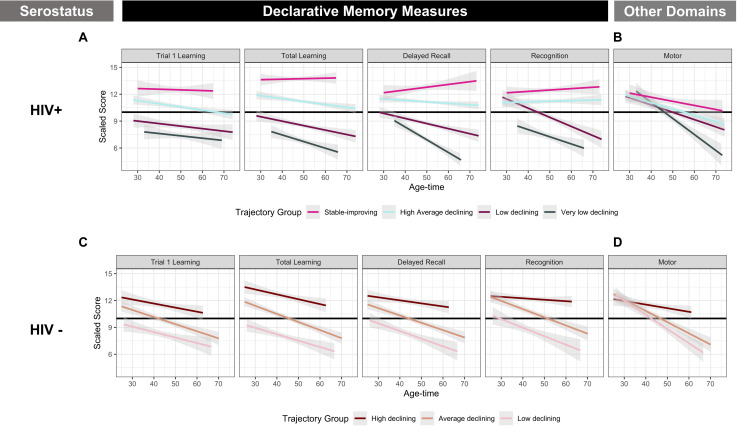
Cluster Groups in White/other WIHS Women. **(A)** Included declarative memory outcomes from the Hopkins Verbal Learning Test-Revised (HVLT-R): memory (delay free recall), learning (total learning), single trial learning (total words recalled on Trial 1), and recognition (number of words correctly identified on a yes/no recognition test). **(B)** Included measures of motor function (time to completion of grooved pegboard test for dominant and non-dominant hands). Values plotted are linear fit of within group averages of scaled averaged across age and time.

We identified four clusters of HIV+ white/other women with significantly different levels of performance at baseline and rates of decline over follow-up (*P*’s < 0.0001; Figure 2A and Table 3). Subgroup specific patterns included a baseline stable/improving group (pink; *n* = 49), a baseline high/average declining (light blue; *n* = 166), a baseline low-declining group (dark purple; *n* = 84) and a very-low-declining group (gray; *n* = 51). While distinct subgroups were identified for declarative memory changes over time, HIV+ white/other women all tended to follow a similar pattern with respect to motor function change over time (*P* for difference in rate of change between clusters = 0.29; Figure 2B). In multivariable-adjusted models, individuals with greater severity of declarative memory decline tended to be of Hispanic ethnicity (*P* = 0.0003), less educated (*P’s* < 0.0001), have lower income (*P* = 0.05) and have poorer viral control (*P* = 0.03). In HIV+VS white/other women (*n* = 94), we identified two clusters with varying levels of baseline performance but generally similar patterns of decline over follow-up (Supplementary Table 4 and Supplementary Figure 2). Similar to larger cohorts of HIV+ white/other women, HIV+ VS white/other women with lower baseline performance tended to be less educated (*P’s <* 0.05), have lower annual household income (*P* = 0.05), and were more likely have a history of depression (*P* = 0.03) and fewer years of ARV (*P* = 0.05) relative to those with higher baseline performance.

**TABLE 3:** Characteristics of HIV-seropositive White/other Women by identified cluster.

**Characteristic**	**Clustering Group**				***P*-value**	
**Group [Color] in Figures 2A,B**	**Stable/improving [pink]**	**High average – declining [light blue]**	**Low – declining [maroon]**	**Very low – declining [gray]**	**Univariate**	Multivariable adjusted*
*N*	49	166	84	49		
Average duration of time intervals between testing, mean (*M*) (Standard deviation [*SD*])	2.2 (0.5)	2.1 (0.4)	2.1 (0.3)	2.2 (0.3)		
Average duration of follow-up, years, *M* (*SD*)	4.6 (2.0)	4.9 (1.7)	4.5 (1.9)	4.8 (1.8)		
Declarative memory at baseline**						
Trial 1 learning, *M* (*SD*)	13.7 (2.3)	10.7 (2.2)	8.9 (2.4)	7.6 (2.4)	<0.0001	**<0.0001**
Total learning, *M* (*SD*)	13.9 (1.8)	11.5 (1.8)	9.2 (2.0)	7.6 (2.2)	<0.0001	**<0.0001**
Delayed recall, *M* (*SD*)	14.6 (1.9)	11.2 (2.1)	8.9 (1.8)	7.3 (2.3)	<0.0001	**<0.0001**
Recognition, *M* (*SD*)	12.5 (2.8)	11.1 (2.7)	9.5 (2.4)	7.0 (1.5)	<0.0001	**<0.0001**
*Rate of change in declarative memory per decade of age-years*						
Trial 1 learning, Beta (B) (95% CI)	0.55 (0.03, 1.06)	−0.36 (−0.59, −0.13)	−0.43 (−0.78, −0.10)	−0.62 (−1.10, −0.62)	0.005	**0.007**
Total learning, B (95% CI)	0.68 (0.15, 1.20)	−0.28 (−0.53, −0.04)	−0.77 (−1.12, −0.42)	−1.21 (−1.71, −0.72)	<0.0001	**<0.0001**
Delayed recall, B(95% CI)	0.41 (−0.12, 0.93)	−0.10 (−0.34, 0.15)	−0.69 (−1.05, −0.34)	−1.47 (−1.97, 0.97)	<0.0001	**<0.0001**
Recognition, B (95% CI)	0.08 (−0.52, 0.69)	0.12 (−0.16, 0.40)	−1.50 (−1.90, −1.10)	−1.18 (−1.86, −0.60)	<0.0001	**<0.0001**
*At initial cognitive test^†^*						
Age, years, *M* (*SD*)	41.6 (7.2)	43.2 (8.7)	42.9 (8.6)	42.6 (7.1)	0.70	0.18
Hispanic ethnicity, *n* (%)	9 (19.2)	61 (36.8)	46 (55.2)	40 (81.6)	<0.0001	**0.0003**
Years of education, *M* (*SD*)	15.5 (2.6)	13.1 (3.1)	11.3 (3.2)	10.1 (3.1)	<0.0001	**0.0004**
WRAT-3 reading, *M* (*SD*)	108.1 (8.9)	101.4 (12.4)	93.1 (17.4)	82.0 (19.2)	<0.0001	**<0.0001**
Annual household income (<$12k/year), *n* (%)	4 (9.3)	50 (33.3)	39 (49.4)	31 (66.0)	<0.0001	**0.05**
Employed, *n* (%)	30 (63.8)	76 (45.8)	27 (32.5)	11 (22.5)	<0.0001	0.78
Depressed^†^, *n* (%)	14 (29.8)	56 (33.9)	56 (32.5)	18 (37.5)	0.88	0.94
Smokes, *n* (%)	8 (17.0)	48 (28.9)	35 (42.2)	28 (57.1)	<0.0001	0.76
Heavy drinker, *n* (%)	3 (6.4)	8 (4.9)	6 (7.2)	3 (6.1)	0.89	0.53
Marijuana use, *n* (%)	8 (17.0)	32 (19.4)	17 (20.5)	4 (8.2)	0.22	0.37
Crack, Cocaine use, *n* (%)	3 (6.4)	7 (4.2)	2 (2.4)	3 (6.1)	0.65	0.17
Heroin use, *n* (%)	1 (2.1)	2 (1.2)	1 (1.2)	1 (2.0)	0.95	0.20
Body mass index, *M* (*SD*)	28.2 (6.5)	28.1 (7.2)	29.8 (8.2)	29.8 (7.3)	0.24	0.84
Hypertension, *n* (%)	15 (31.9)	58 (34.9)	27 (32.5)	15 (30.6)	0.94	0.39
Diabetes, *n* (%)	6 (12.8)	31 (18.7)	18 (21.7)	15 (30.6)	0.16	0.48
Hepatitis C RNA positive, *n* (%)	1 (2.1)	27 (16.3)	18 (21.7)	9 (18.4)	0.007	0.08
Years of ARV, *n* (%)	11.7 (4.8)	11.8 (4.5)	11.8 (4.6)	11.2 (5.5)	0.89	0.31
Years of HAART, *n* (%)	10.3 (4.4)	10.3 (4.2)	9.7 (4.4)	9.7 (5.1)	0.65	0.25
CD4 count < 200, *n* (%)	1 (2.1)	18 (11.0)	9 (10.8)	8 (16.3)	0.08	0.39
HIV RNA < 48 cp/mL, *n* (%)	30 (63.8)	93 (56.4)	52 (62.7)	19 (38.8)	0.04	**0.02**
HIV RNA > 10,000 cp/mL, *n* (%)	3 (6.4)	14 (8.5)	8 (9.6)	6 (12.2)	0.78	0.44
*% of WIHS visits prior to cognitive testing^‡^*						
Annual household income (<$12k/year), *M* (*SD*)	16.5 (27.8)	33.6 (33.8)	48.9 (34.2)	59.3 (34.1)	<0.0001	**0.04**
Employed, *M* (*SD*)	62.0 (33.2)	44.7 (36.5)	33.2 (33.8)	27.4 (35.5)	<0.0001	0.28
Depressed, *M* (*SD*)	28.2 (30.5)	36.5 (30.1)	41.1 (32.3)	50.3 (34.8)	0.0006	0.64
Smokes, *M* (*SD*)	18.6 (34.1)	35.1 (39.7)	42.5 (42.2)	56.0 (42.4)	<0.0001	0.81
Heavy drinker, *M* (*SD*)	2.7 (8.0)	2.4 (7.7)	3.0 (8.9)	5.0 (11.9)	0.35	0.32
Marijuana use, *M* (*SD*)	13.8 (27.0)	17.7 (28.7)	22.4 (34.0)	12.1 (24.6)	0.19	0.29
Crack, Cocaine use, *M* (*SD*)	4.1 (13.4)	4.6 (15.3)	6.0 (15.3)	7.7 (19.0)	0.59	0.64
Heroin use, *M* (*SD*)	2.4 (13.7)	1.8 (9.1)	2.6 (10.0)	2.8 (10.7)	0.91	0.05
CD4 count < 200, *M* (*SD*)	6.6 (14.0)	11.5 (19.2)	12.7 (21.2)	15.8 (23.0)	0.14	0.48
HIV RNA < 48 cp/mL, *M* (*SD*)	11.7 (17.4)	8.0 (12.2)	12.3 (20.6)	5.3 (6.2)	0.02	**0.003**
HIV RNA > 10,000 cp/mL, *M* (*SD*)	16.7 (17.7)	17.4 (18.4)	21.3 (22.2)	22.8 (25.8)	0.23	0.16

*^∗^Adjusted for all factors included in the table. ^∗∗^Values presented are scaled to have a mean of 10 and a standard deviation of 3. ^†^Calculated as the baseline (at initial cognitive visit) exposure level. ^‡^Calculated as a cumulative exposure level defined as the percentage of visits prior to cognitive testing in which the individual was ‘exposed’ (e.g., if an individual reports a current smoker status for 10 of 30 visits, the cumulative exposure level is defined as 0.30). M, mean; SD, standard deviation; B, beta coefficient; CI, confidence interval; HAART, highly active antiretroviral therapy; ARV, antiretroviral. Bolded p-values denote those which are < 0.05 in multivariable-adjusted models.*

### HIV− White/Other Women

We identified three clusters of HIV− white/other women with distinct levels of baseline performance and rates of decline over follow-up (Figure 2C and Table 4). Notably, all of the identified subgroups tended to have a similar pattern of change with respect to motor function, despite having distinct patterns with respect to declarative memory (*P* for difference in rate of change in motor function between clusters = 0.14; Figure 2D). Subgroup specific patterns included a baseline high-declining group (maroon; *n* = 57), an average-declining group (salmon; *n* = 71) and a low-declining group (pink; *n* = 51). In multivariable models, individuals in the average-declining and low-declining groups were less educated (Table 4; *P* = 0.003) and were less likely to have a history of marijuana use (*P* = 0.04) versus those in the high-declining subgroups.

**TABLE 4:** Characteristics of HIV-seronegative White/other Women by identified cluster.

**Characteristic**	**Clustering Group**			***P*-value**	
**Group [Color] in Figures 2C,D**	**High – declining [reddish brown]**	**Average – declining [salmon]**	**Low – declining [light pink]**	**Univariate**	**Multivariable adjusted**
*N*	57	77	51		
Average duration of time intervals between testing, mean (*M*) (Standard deviation [*SD*])	2.2 (0.3)	2.2 (0.6)	2.2 (0.6)		
Average duration of follow-up, *M* (*SD*)	4.8 (1.8)	4.0 (2.0)	4.1 (1.8)		
Declarative memory at baseline**					
Trial 1 learning, *M* (*SD*)	12.8 (2.6)	10.4 (2.7)	8.6 (2.6)	<0.0001	**<0.0001**
Total learning, *M* (*SD*)	13.3 (2.0)	10.7 (2.2)	8.8 (2.6)	<0.0001	**<0.0001**
Recognition, *M* (*SD*)	12.2 (1.8)	10.9 (2.3)	8.3 (2.9)	<0.0001	**<0.0001**
Delayed recall, *M* (*SD*)	13.3 (2.2)	10.8 (2.0)	8.0 (2.3)	<0.0001	**<0.0001**
*Rate of change in declarative memory per decade of age-years*					
Trial 1 learning, Beta (B) (95% CI)	−0.74 (−1.23, −0.25)	−1.13 (−1.49, −0.78)	−0.75 (−1.32, −0.19)	0.27	0.08
Total learning, B (95% CI)	−0.30 (−0.75, −0.15)	−1.06 (−1.40, −0.73)	−0.85 (−1.35, −0.33)	0.04	0.001
Delayed recall, B (95% CI)	−0.52 (−1.00, −0.04)	−1.06 (−1.43, −0.70)	−0.75 (−1.30, −0.23)	0.20	0.09
Recognition, B (95% CI)	−0.16 (−0.53, 0.21)	−0.85 (−1.10, −0.60)	−0.90 (−1.34, −0.46)	0.006	0.001
*At initial cognitive test^†^*					
Age, years, *M* (*SD*)	35.9 (8.7)	38.0 (12.4)	37.2 (8.9)	0.53	0.48
Hispanic ethnicity, *n* (%)	25 (43.9)	51 (66.23)	33 (64.7)	0.02	0.09
Years of education, *M* (*SD*)	14.0 (3.1)	12.5 (2.7)	10.6 (2.7)	<0.0001	**0.003**
WRAT-3 reading, *M* (*SD*)	102 (14.9)	95.9 (17.3)	83.3 (17.6)	<0.0001	0.21
Annual household income (<$12k/year), *n* (%)	13 (25.0)	34 (46.0)	33 (67.4)	<0.0001	0.11
Employed, *n* (%)	13 (25.0)	34 (46.0)	33 (67.4)	0.0006	0.61
Depressed^†^, *n* (%)	11 (19.3)	30 (39.5)	19 (37.3)	0.03	0.14
Smokes, *n* (%)	23 (40.4)	34 (44.2)	27 (52.9)	0.41	0.34
Heavy drinker, *n* (%)	5 (8.9)	8 (10.4)	5 (9.8)	0.95	0.34
Marijuana use, *n* (%)	17 (29.8)	19 (24.7)	7 (13.7)	0.12	0.63
Crack, Cocaine use, *n* (%)	5 (8.8)	6 (7.8)	2 (3.9)	0.55	0.51
Heroin use, *n* (%)	1 (1.8)	2 (2.6)	2 (3.9)	0.79	0.84
Body mass index, *M* (*SD*)	31.5 (6.9)	31.9 (7.6)	31.8 (7.5)	0.95	0.62
Hypertension, *n* (%)	15 (26.3)	25 (32.5)	19 (37.3)	0.47	0.64
Diabetes, *n* (%)	9 (15.8)	18 (23.4)	12 (23.5)	0.49	0.99
Hepatitis C RNA positive, *n* (%)	7 (12.3)	7 (9.1)	6 (11.8)	0.81	0.69
*% of WIHS visits prior to cognitive testing^‡^*					
Annual household income (<$12k/year), *M* (*SD*)	24.3 (25.0)	45.5 (32.9)	55.2 (31.2)	<0.0001	0.55
Employed, *M* (*SD*)	64.0 (32.2)	45.1 (32.9)	31.2 (30.3)	<0.0001	0.18
Depressed, *M* (*SD*)	22.2 (25.1)	31.1 (28.4)	38.9 (29.5)	0.008	0.97
Smokes, *M* (*SD*)	45.3 (38.4)	48.7 (39.7)	57.5 (42.7)	0.27	0.59
Heavy drinker, *M* (*SD*)	5.1 (13.8)	9.8 (19.4)	12.9 (20.1)	0.25	0.45
Marijuana use, *M* (*SD*)	34.7 (35.6)	28.2 (34.2)	20.1 (27.1)	0.07	**0.04**
Crack, Cocaine use, *M* (*SD*)	6.0 (13.3)	15.5 (26.1)	11.4 (21.0)	0.04	0.20
Heroin use, *M* (*SD*)	5.7 (18.4)	6.3 (16.5)	6.5 (16.0)	0.96	0.76

*^∗^Adjusted for all factors included in the table.^∗∗^Values presented are scaled to have a mean of 10 and a standard deviation of 3. ^†^Calculated as the baseline (at initial cognitive visit) exposure level. ^‡^Calculated as a cumulative exposure level defined as the percentage of visits prior to cognitive testing in which the individual was ‘exposed’ (e.g., if an individual reports a current smoker status for 10 of 30 visits, the cumulative exposure level is defined as 0.30). M, mean; SD, standard deviation; B, beta coefficient; CI, confidence interval. Bolded p-values denote those which are < 0.05 in multivariable-adjusted models.*

## Discussion

Here, we leveraged a rich longitudinal dataset including large groups of both virally suppressed and non-virally suppressed women to examine longitudinal phenotyping of declarative memory, a domain commonly impaired, among HIV+ compared to HIV− women (Maki et al., 2015; Rubin and Maki, 2019). We advanced our previous findings in women with HIV by disentangling the heterogeneity in declarative memory over age-time among HIV+ and HIV− women (Rubin et al., 2017b) using a data driven modeling approach. Furthermore, we also found that in order to separate moderately independent clusters of women with similar patterns of change in declarative memory, stratification by race was necessary. In general, we determined that among Black/African American and White/Other HIV+ and HIV− women, the subgroups identified showed initial differences in declarative memory performance across specific tests. We also observed different subgroups within HIV+ and HIV− women with different patterns of decline, which is important, as the rate of change in declarative memory was not substantially different when considering HIV+ and HIV− groups globally. There were also possible differences in age-related (as age was the time scale in our analyses) rates of change in declarative within each race stratum even though there were no age differences between subgroups at the initial neuropsychological testing visit. Notably, while we observed general patterns of decline in declarative memory among HIV- women, we did not find evidence of significant decline among HIV+VS women. The lack of a decline among HIV+VS women provides possible support for the effectiveness of ART. With that being said, the sample size was also substantially smaller for the HIV+VS, so it’s also possible that the lack of significance may stem from a lack of power to detect an effect. Additionally, changes in declarative memory within subgroups did not necessarily track with other cognitive domains of motor function, suggesting composite measures of cognitive function incorporating multiple domains may mask key differences within the population or could dilute beneficial or adverse effects of candidate prognostic factors.

Analyses indicated that the most common predictor that distinguished subgroups within HIV-serostatus and race strata was fewer years of education and lower educational attainment measured via the WRAT-3 reading subscale. Across all serostatus strata and racial groups, suboptimal educational experience increased susceptibility to poorer declarative memory profiles, suggesting that cognitive reserve may be universally protective against such declines, which is relatively consistent with previous research (Baker et al., 1998; Manly et al., 2003, 2011). We included women with low verbal IQ (e.g., WRAT-3 < 85) in our analyses, and the assessment of verbal memory in individuals with low verbal IQs using standard assessments is potentially problematic. However, as women with HIV living in the United States are disproportionately likely to also have low socio-economic status, low levels of education, and a history of substance use, we included these individuals so to facilitate interpretation of our results in the context of HIV.

Notably, a key finding of our study suggests that different non-HIV status predictors distinguish subgroups across strata (e.g., HIV+ Black/African American, HIV+ White/Other, HIV− Black/African American, HIV− White/Other). For example, within HIV+ Black/African American women, the strongest predictors differentiating subgroups included unemployment, history of depression, vascular and metabolic factors including obesity and diabetes, as well as a history of crack, cocaine, and heroin use in virally suppressed women. Numerous studies have demonstrated that these factors are negatively associated with aspects of declarative memory in PWH. For example, we have previously shown in the WIHS that HIV+ recent crack/cocaine and/or heroin users compared to HIV+ non-users performed lower on total learning and delayed free recall of the HVLT (Meyer et al., 2013). Depression is also negatively associated with these two outcomes in the WIHS (Rubin et al., 2014; Maki et al., 2015). Our observation that individuals with more severe declarative memory declines were less likely to be obese and have diabetes was an unexpected finding. It’s possible that individuals in these groups were more likely to have a greater burden of physical impairments (and relatedly, possibly reduced muscle mass and weight).

In contrast to Black/African American HIV+ women, vascular and metabolic factors (e.g., obesity, diabetes, and hypertension) and substance use did not emerge as predictors of trajectory membership in HIV+ White/Other women. While the prevalence of diabetes and current marijuana use was similar among HIV+ Black/African American (19 and 17%, respectively) and HIV+ White/Other women (20 and 18%), Black/HIV+ African American women had reported a greater lifetime crack/cocaine use and were slightly more obese (*M* = 31, *SD* = 8) compared to HIV+ White/Other women (*M* = 29, *SD* = 7). Among HIV+ White/Other women, factors emerging as predictors of trajectory membership included being Hispanic, lower annual household income, and viral control, as well as history of depression in virally suppressed women predicted group membership. Viral control is known to be an important predictor of declarative memory performance. For example, we previously demonstrated that HIV+ women with intermittent combination ART use and inconsistent plasma viral suppression showed initial differences in total learning on the HVLT that persisted over a 4 years duration compared to HIV+VS women (Rubin et al., 2017b). Women with inconsistent viral suppression also demonstrated initial differences in delayed recall on the HVLT that also persisted over a 4-year duration compared to HIV+ women with consistent use of combination ART but inconsistent plasma viral suppression. Collectively, our analyses highlight the importance of race as a contributor to individual differences in memory and suggests that key social determinants associated with race in the United States (e.g., education level, access to healthcare and poverty) may be critical drivers of these differences.

HIV− WIHS women are comparable to HIV+ WIHS women with respect to ethnic composition, socioeconomic status (including education status), substance use, and comorbidities (e.g., depression). This representative nature of the HIV− group is notable as these women also demonstrated a decline in scores and were at an increased risk of NCI (similar to that which was observed in the HIV+ women). If the population of HIV− women had been healthier (and not as representative), we would expect the WIHS HIV− to demonstrate stable or improving (related to practice effects) neuroperformance over the course of the study. On a similar note, we also highlight that HIV− WIHS participants demonstrate on average 2 SD below age-adjusted norms for woman of comparable ages on learning and memory (Benedict et al., 1998; Norman et al., 2011; Rubin et al., 2017b).

Among HIV− Black/African American and White/Other women, illicit substance use was a common predictor across strata, although the substance of preference differed. Current and history of crack/cocaine use predicted subgroup membership (susceptibility to poorer declarative memory profiles) among HIV− Black/African American women whereas marijuana use predicted subgroup membership (susceptibility to better declarative memory profiles) among HIV− White/Other women. Both illicit substances have been adversely associated with memory performance (Verdejo-García et al., 2006; Fernández-Serrano et al., 2011). Previous studies consistently link crack/cocaine with poor neuropsychological performance across a number of domains, including declarative memory (Meyer et al., 2013; Lopes et al., 2017). Studies of the non-acute effects of marijuana on cognition yield mixed results. A recent meta-analyses suggested effect size of marijuana use on cognitive function may be of little clinical importance, so it’s possible that our finding of marijuana being associated better declarative memory profiles in White/Other women may be a chance finding (Scott et al., 2018).

There are a few study limitations. Since we required at least two complete neuropsychological assessments to meet eligibility for analysis, loss to follow-up is one limitation. Further, we fit linear models for all trajectories when it is possible that incorporating more complex functions of time are appropriate; however, as women had on average three neuropsychological assessments (making more complex functions of time more difficult), we chose to be conservative and use only linear trajectories. Follow-up studies will incorporate polynomial and spline functions of time. Missingness may be non-random; however, missingness was relatively rare as ∼99% of declarative memory tests were complete. Second, we only focused on baseline/initial or pre-baseline risk factors and thus we are not considering the effect of initiation or cessation of poor/good health behavior. Third, although we examined depression as a predictor of cognition, declarative memory issues may have led to depression. Fourth, we only crudely adjusted for HIV treatment/adherence. Specific ART therapies may have differential effects on declarative memory profiles. While outside of the scope of the present manuscript, we will be examining this issue in subsequent analyses. We also acknowledge that it’s possible initial starting value may have been impacted by an individual’s cognitive reservoir (which may also be associated with race and education status); such reserve may have a stronger influence on cognition than the initial adverse effects of HIV on cognition. Lastly, our study was limited in that we also could not evaluate how memory trajectories were related to activities of daily living as this questionnaire (e.g., Instrumental activities of daily living; IADL) was not asked in WIHS until later in follow-up. It’s possible that memory trajectory could be a key determinant of everyday functioning.

Our analyses employed a Bayesian approach to identify longitudinal clusters of women using a Dirichlet process prior distribution. We highlight that this analysis serves as one example of an approach to define multivariate clusters of longitudinal data. Other statistical methods including those described by Genolini et al. (2015) and Proust-Lima et al. (2017) were developed to perform similar functions but employing different underlying clustering methodologies and highlight the relative importance of considering both latent heterogeneity and multivariate longitudinal data collectively, as in this analysis.

In sum, we employed a data-driven modeling approach (of several other approaches with similar goals) that successfully identified meaningful subgroups of individuals with distinct phenotypes of declarative memory decline that did not mirror changes in motor function measures. Among the different groups of HIV+ and HIV− Black/African American versus White/Other women, we identified a number of factors that helped to determine subgroup membership. While some factors are not modifiable or varied across subgroups, depression among HIV+ Black/African American women and HIV+VS White/other women was identified as a key, possibly modifiable determinant of membership in a subgroup characterized by more rapid decline. Consequently, mental health assessment and potential antidepressant treatment should remain at the forefront of cognitive sequela in women with HIV. Further, our results suggest that consideration of both HIV serostatus as well as race are an important component in understanding the evolution of cognitive impairment in such populations. Lastly, we note that much of the underlying heterogeneity in cognitive trajectories remained was unexplained by participant characteristics or routinely measured clinical risk factors. Thus, our study sets the stage for future research that aims to disentangle underlying candidate biological mechanisms or measure more proximal intermediates (e.g., genetic, blood- or imaging-based markers) that drive the observed clusters cluster membership.

## Data Availability Statement

Data from the WIHS study is collected as a part of a long-term research project and is not publicly available; access to de-identified subject level from WIHS is controlled by an established governance structure and can only be granted through collaborations with existing WIHS investigators.

## Ethics Statement

The studies involving human participants were reviewed and approved by Johns Hopkins Institutional Review Board. The patients/participants provided their written informed consent to participate in this study.

## Author Contributions

LR and KF conceived the study idea and wrote the first draft of the manuscript. KF also took responsibility for the integrity of the analyses. All the authors contributed to the writing of the manuscript and approved the final version of the article.

## Conflict of Interest

The authors declare that the research was conducted in the absence of any commercial or financial relationships that could be construed as a potential conflict of interest.
