# Supplementary material for: Factors Predicting Detrimental Change in Declarative Memory Among Women With HIV: A Study of Heterogeneity in Cognition
Source: Front Psychol. 2020 Oct 15;11:548521. doi: 10.3389/fpsyg.2020.548521 (PMC7594511; doi:10.3389/fpsyg.2020.548521)
Supplement: Supplementary file 1 [file Table_1.docx]

**Supplemental Table 1. Overall Characteristics of HIV-seropositive (HIV+) and HIV-seronegative (HIV-) WIHS Study Participants**

|  | **African American WIHS Women** | | | |  | **White/other WIHS Women** | | | |
| --- | --- | --- | --- | --- | --- | --- | --- | --- | --- |
|  | **Black/African American women** | | | |  | **White/Other women** | | | |
|  | **All HIV+** | **HIV+ VS** | **HIV-** | **P value HIV+ vs. HIV-** |  | **All HIV+** | **HIV+ VS** | **HIV-** | **P value for HIV+ vs. HIV-** |
| N | 804 | 166 | 397 |  |  | 348 | 94 | 185 |  |
| *Declarative memory at baseline* |  |  |  |  |  |  |  |  |  |
| Trial 1 learning, M (SD) | 9.8 (2.9) | 10.2 (2.9) | 10.3 (2.9) | **0.01** |  | 10.3 (2.9) | 10.5 (3.1) | 10.6 (3.1) | 0.16 |
| Total learning, M (SD) | 9.7 (2.8) | 9.9 (2.8) | 10.3 (3.0) | **0.001** |  | 10.7 (2.7) | 10.6 (2.9) | 11.0 (2.8) | 0.48 |
| Delayed recall, M (SD) | 9.6 (2.8) | 10.1 (2.6) | 10.3 (3.0) | **<0.001** |  | 10.6 (3.0) | 10.6 (3.5) | 10.8 (3.0) | 0.33 |
| Recognition, M (SD) | 9.6 (3.0) | 9.7 (2.9) | 9.8 (3.0) | 0.22 |  | 10.4 (2.9) | 10.1 (3.1) | 10.6 (2.8) | 0.45 |
| *Rate of change in declarative memory per decade of age-years* |  |  |  |  |  |  |  |  |  |
| Trial 1 learning, Beta (B) (95% CI) | -0.50 (-0.67, -0.33) | -0.22 (-0.63, 0.20) | -0.41 (-0.62, -0.21) | 0.47 |  | -0.49 (-0.81, -0.17) | -0.30 (-1.00, 0.42) | -1.00 (-1.33, -0.67) | **0.03** |
| Total learning, B (95% CI) | -0.53 (-0.70, -0.37) | -0.46 (-0.87, -0.07) | -0.67 (-0.90, -0.43) | 0.57 |  | -0.43 (-0.72, -0.13) | -0.12 (-0.82, -0.58) | -0.92 (-1.27, -0.58) | 0.13 |
| Delayed recall, B (95% CI) | -0.57 (-0.74, -0.40) | -0.36 (-0.76, 0.02) | -0.51 (-0.74, -0.29) | 0.42 |  | -0.54 (-0.86, -0.23) | -0.15 (-0.89, 0.60) | -0.82 (-1.27, -0.57) | **0.04** |
| Recognition, B (95% CI) | -0.51 (-0.69, -0.34) | -0.51 (-0.89, -0.13) | -0.43 (-0.65, -0.21) | 0.56 |  | -0.51 (-0.22, -0.80) | -0.19 (-0.82, 0.44) | -0.74 (-1.02, -0.45) | 0.18 |
| *At initial cognitive test†* |  |  |  |  |  |  |  |  |  |
| Age, years, M (SD) | 41.8 (8.5) | 42.6 (7.9) | 40.2 (10.2) | **0.006** |  | 42.8 (8.3) | 43.6 (7.4) | 37.1 (10.4) | **<0.0001** |
| Hispanic ethnicity | 25 (3.2) | 3 (1.9) | 18 (4.7) | 0.28 |  | 156 (45.2) | 36 (38.7) | 109 (58.9) | **0.002** |
| Years of education, M (SD) | 12.4 (2.8) | 12.5 (3.0) | 12.6 (2.9) | 0.32 |  | 12.6 (3.4) | 13.1 (3.3) | 12.5 (3.1) | 0.72 |
| WRAT-3 reading, M (SD) | 89.4 (18.0) | 89.2 (17.8) | 89.7 (17.1) | 0.59 |  | 97.6 (16.4) | 99.8 (15.2) | 94.4 (18.1) | **0.05** |
| Annual household income (<$12k/year) | 385 (51.8) | 67 (43.2) | 186 (52.1) | 0.86 |  | 124 (38.9) | 25 (28.4) | 80 (45.7) | 0.18 |
| Employed | 261 (33.0) | 72 (44.4) | 165 (42.9) | **0.0007** |  | 144 (417.7) | 46 (49.5) | 79 (42.7) | 0.68 |
| Depressed | 242 (30.7) | 47 (29.0) | 111 (28.8) | 0.41 |  | 115 (33.5) | 28 (30.1) | 60 (32.6) | 0.68 |
| Smokes | 337 (42.7) | 52 (32.1) | 188 (48.8) | **0.04** |  | 119 (34.5) | 21 (22.6) | 84 (45.4) | **0.01** |
| Heavy drinker | 52 (6.6) | 6 (3.7) | 48 (12.5) | **0.001** |  | 20 (5.8) | 1 (1.1) | 18 (9.7) | 0.10 |
| Marijuana use | 131 (16.6) | 22 (13.6) | 84 (24.4) | **0.002** |  | 61 (17.7) | 10 (10.8) | 43 (23.2) | 0.15 |
| Crack, Cocaine use | 50 (6.3) | 4 (2.5) | 41 (10.7) | **0.006** |  | 15 (4.4) | 1 (1.1) | 13 (7.0) | 0.20 |
| Heroin use | 12 (1.5) | 2 (1.2) | 7 (1.8) | 0.20 |  | 5 (1.5) | 0 (0.00) | 5 (2.7) | 0.47 |
| Body mass index, M (SD) | 30.8 (8.4) | 31.3 (8.8) | 32.6 (9.0) | **0.0007** |  | 28.8 (7.4) | 27.8 (7.1) | 31.7 (7.3) | **<0.0001** |
| Hypertension | 371 (47.0) | 79 (48.8) | 166 (43.1) | 0.22 |  | 115 (33.3) | 36 (38.7) | 59 (31.9) | 0.59 |
| Diabetes | 151 (19.1) | 38 (23.5) | 77 (20.0) | 0.76 |  | 70 (20.3) | 23 (24.7) | 39 (21.1) | 0.89 |
| Hepatitis C RNA positive | 146 (18.5) | 25 (15.6) | 40 (10.4) | **0.0001** |  | 55 (15.9) | 17 (18.3) | 20 (10.8) | 0.10 |
| Years of ARV | 9.4 (5.4) | 10.8 (4.9) | - |  |  | 10.1 (4.4) | 12.5 (4.2) | - |  |
| Years of HAART | 8 (4.7) | 9.2 (4.2) | - |  |  | 11.7 (4.7) | 10.5 (4.3) | - |  |
| CD4 count <200 | 88 (11.2) | 3 (1.9) | - |  |  | 36 (10.5) | 5 (5.4) | - |  |
| HIV RNA <48cp/mL | 426 (54.3) | 156 (96.3) | - |  |  | 194 (56.4) | 86 (92.5) | - |  |
| HIV RNA > 10,000 cp/mL | 102 (13.0) | 0 (0.00) | - |  |  | 31 (9.0) | 0 (0.0) | - |  |
| *% of WIHS visits prior to cognitive testing* |  |  |  |  |  |  |  |  |  |
| Annual household income (<$12k/year) | 50.9 (36.2) | 44.0 (36.0) | 49.4 (35.8) | **0.04** |  | 38.6 (35.5) | 30.8 (33.9) | 41.6 (32.7) | **0.005** |
| Employed | 34.4 (36.6) | 44.6 (38.7) | 41.6 (35.2) | **0.05** |  | 41.9 (36.6) | 52.3 (38.7) | 47.1 (34.2) | 0.49 |
| Depressed | 37.1 (33.5) | 32.3 (32.9) | 34.8 (31.7) | 0.60 |  | 36.3 (32.0) | 33.2 (34.1) | 30.5 (28.3) | **0.05** |
| Smokes | 46.4 (43.4) | 37.9 (42.5) | 54.4 (43.9) | 0.06 |  | 37.7 (41.1) | 24.8 (35.9) | 50.1 (40.3) | **<0.0001** |
| Heavy drinker | 7.2 (17.9) | 4.7 (14.5) | 11.7 (21.9) | 0.94 |  | 2.9 (8.8) | 2.1 (5.9) | 7.7 (16.2) | 0.98 |
| Marijuana use | 17.4 (29.7) | 13.0 (27.3) | 28.3 (35.0) | **0.001** |  | 17.5 (29.4) | 9.2 (22.1) | 28.0 (33.2) | 0.93 |
| Crack, Cocaine use | 10.4 (23.2) | 6.3 (19.3) | 16.2 (28.0) | 0.12 |  | 5.3 (15.6) | 1.9 (6.4) | 11.4 (21.7) | 0.58 |
| Heroin use | 3.1 (12.6) | 2.6 (14.1) | 3.8 (13.9) | 0.84 |  | 2.2 (10.2) | 1.1 (8.0) | 6.2 (16.9) | **0.006** |
| CD4 count <200 | 11.1 (20.6) | 6.7 (13.4) | - |  |  | 11.7 (19.8) | 8.3 (18.6) | - |  |
| HIV RNA <48cp/mL | 13.0 (24.6) | 23.2 (30.5) | - |  |  | 9.2 (15.0) | 13.6 (17.4) | - |  |
| HIV RNA > 10,000 cp/mL | 21.5 (24.3) | 10.7 (13.8) | - |  |  | 19.0 (20.5) | 11.5 (14.6) | - |  |

*Calculated as a cumulative exposure level defined as the percentage of visits prior to cognitive testing in which the individual was ‘exposed’ (e.g. if an individual reports a current smoker status for 10 of 30 visits, the cumulative exposure level is defined as 0.30). M=mean; SD=standard deviation; B=beta coefficient; CI=confidence interval; HAART=highly active antiretroviral therapy; ARV=antiretroviral. †Denotes significant difference (p-value <0.05) when comparing a given characteristic between HIV+ vs. HIV- Black women. ‡Bolded values denotes significant difference (p-value <0.05) when comparing a given characteristic between HIV+ vs. HIV- White/other women.

**Supplemental Table 2. Summary of Trajectory-based analyses***

|  |  | | | | |  | |
| --- | --- | --- | --- | --- | --- | --- | --- |
| Racial Group | **Black/African American** | | | **White/Other** | | | |
|  | **HIV+** | **HIV-** | **HIV+VS** | **HIV+** | **HIV-** | | **HIV+VS** |
| Number of identified clusters | 4 | 4 | 3 | 4 | 3 | | 2 |
| Total N  (Number participants per cluster) | 804  (99; 340; 267; 95) | 397  (48; 150; 148; 51) | 166  (37; 89; 40) | 348  (49; 166; 84; 49) | 185  (57; 77; 51) | | 94 (29 \| 65) |
| Table/  Figure describing results | Table 1/ Figure 1A-B | Table 2/ Figure 1C-D | Supplemental Table 3; Supplemental Figure 1 | Table 3/ Figure 2A-B | Table 4/ Figure 2C-D | | Supplemental Table 4; Supplemental Figure 1 |

*For each strata, we compared individual declarative memory tests (4 tests), motor function, and selected demographic, clinical, HIV, and lifestyle characteristics both at baseline (21 characteristics) and occurring over follow-up (11 characteristics)

**Supplemental Table 3. Characteristics of Virally Suppressed HIV-seropositive Black/African American Women by Identified Baseline + Trajectory Clustering Group.**

| **Characteristic** | **Trajectory Group** | | | **P-value** | |
| --- | --- | --- | --- | --- | --- |
| Group [Color] in Supplemental Figure 1 | **High declining**  **[dark blue]** | **Average declining**  **[medium blue]** | **Low declining**  **[light blue]** | **Univariate** | **Multivariable Adjusted*** |
| N | 37 | 89 | 40 |  |  |
| Average duration of time intervals between testing, mean (M) (Standard deviation [SD]) | 2.1 (0.2) | 2.1 (0.6) | 2.2 (0.6) |  |  |
| Average duration of follow-up, years, M (SD) | 5.0 (1.6) | 4.5 (1.9) | 4.6 (1.8) |  |  |
| *Declarative memory at baseline* |  |  |  |  |  |
| Trial 1 learning, M (SD) | 13.18 (2.59) | 10.16 (2.24) | 7.75 (1.99) | <0.0001 | **<0.0001** |
| Total learning, M (SD) | 12.79 (1.82) | 10.06 (2.13) | 7.00 (1.90) | <0.0001 | **<0.0001** |
| Recognition, M (SD) | 11.80 (1.77) | 9.90 (2.65) | 7.43 (2.59) | <0.0001 | **<0.0001** |
| Delayed recall, M (SD) | 13.04 (2.07) | 10.13 (1.68) | 7.41 (1.65) | <0.0001 | **<0.0001** |
| *Rate of change in declarative memory per decade of age-years* |  |  |  |  |  |
| Trial 1 learning, Beta (B) (95% CI) | -0.82 (-1.59, -0.03) | -0.31 (-0.75, 0.12) | -0.21 (-0.92, 0.50) | 0.55 | 0.29 |
| Total learning, B (95% CI) | -0.66 (-1.30, -0.01) | -0.72 (-1.09, -0.36) | -0.44 (-1.04, 0.14) | 0.71 | 0.91 |
| Delayed recall, B (95% CI) | -0.53 (-1.18, 0.11) | -0.48 (-0.85, -0.11) | -0.56 (-1.14, 0.03) | 0.97 | 0.84 |
| Recognition, B (95% CI) | -0.09 (-0.74, 0.55) | -0.81 (-1.23, -0.59) | -0.81 (-1.41, -0.21) | 0.08 | 0.28 |
| *At initial cognitive test†* |  |  |  |  |  |
| Age, years, M (SD) | 41.9 (6.6) | 43.4 (8.6) | 41.5 (6.9) | 0.38 | 0.15 |
| Hispanic ethnicity, n (%) | 1 (2.9) | 1 (1.1) | 1 (2.6) | 0.74 | 0.93 |
| Years of education, M (SD) | 14.2 (2.9) | 12.5 (3.2) | 11.1 (2.0) | <0.0001 | **0.01** |
| WRAT-3 reading, M (SD) | 96.9 (15.4) | 90.2 (16.6) | 80.4 (19.2) | 0.0002 | **0.02** |
| Annual household income (<$12k/year), n  (%) | 9 (27.3) | 36 (42.4) | 22 (59.5) | 0.02 | 0.98 |
| Employed, n (%) | 23 (67.7) | 42 (47.2) | 7 (18.0) | <0.0001 | **0.02** |
| Depressed^†^, n (%) | 5 (17.7) | 27 (30.3) | 15 (38.5) | 0.06 | 0.39 |
| Smokes, n (%) | 8 (23.5) | 27 (30.3) | 17 (436) | 0.17 | 0.48 |
| Heavy drinker, n (%) | 3 (8.8) | 3 (3.4) | 0 (0.0) | 0.09 | 0.03 |
| Marijuana use, n (%) | 4 (11.8) | 11 (12.4) | 7 (18.0) | 0.67 | 0.91 |
| Crack, Cocaine use, n (%) | 0 (0.0) | 1 (1.1) | 3 (7.7) | 0.06 | 0.09 |
| Heroin use, n (%) | 0 (0.0) | 1 (1.1) | 1 (2.6) | 0.53 | 0.23 |
| Body mass index, M (SD) | 32.6 (9.0) | 31.4 (9.5) | 30.1 (6.7) | 0.49 | 0.61 |
| Hypertension, n (%) | 19 (55.9) | 44 (49.4) | 16 (41.0) | 0.44 | 0.87 |
| Diabetes, n (%) | 11 (32.4) | 21 (23.6) | 6 (15.4) | 0.23 | 0.83 |
| Hepatitis C RNA positive, n (%) | 5 (14.7) | 18 (20.5) | 2 (5.3) | 0.06 | 0.06 |
| Years of ARV, M (SD) | 10.8 (5.4) | 11.1 (4.6) | 10.0 (5.1) | 0.53 | 0.06 |
| Years of HAART, M (SD) | 8.9 (5.0) | 9.5 (3.8) | 9.0 (4.2) | 0.73 | 0.27 |
| CD4 count <200, n (%) | 0 (0.0) | 1 (1.1) | 2 (5.1) | 0.21 | 0.66 |
| *% of WIHS visits prior to cognitive testing*** |  |  |  |  |  |
| Annual household income (<$12k/year), M  (SD) | 29.1 (27.1) | 43.4 (35.8) | 38.2 (58.5) | 0.002 | 0.11 |
| Employed, M (SD) | 64.9 (31.1) | 48.6 (38.3) | 30.8 (17.7) | <0.0001 | **<0.0001** |
| Depressed, M (SD) | 19.8 (25.1) | 29.5 (30.4) | 49.6 (37.8) | 0.0002 | 0.41 |
| Smokes, M (SD) | 34.7 (43.2) | 34.4 (41.1) | 48.7 (44.4) | 0.19 | 0.76 |
| Heavy drinker, M (SD) | 3.5 (11.5) | 2.6 (8.4) | 10.3 (23.8) | 0.02 | 0.14 |
| Marijuana use, M (SD) | 13.8 (30.8) | 9.9 (23.0) | 19.5 (32.2) | 0.18 | 0.78 |
| Crack, Cocaine use, M (SD) | 1.2 (3.0) | 5.8 (17.7) | 11.9 (28.1) | 0.06 | **0.03** |
| Heroin use, M (SD) | 0.0 (0.0) | 1.6 (10.8) | 6.9 (23.4) | 0.07 | **0.03** |

*Adjusted for all factors included in the table. †Calculated as the baseline (at initial cognitive visit) exposure level. **Calculated as a cumulative exposure level defined as the percentage of visits prior to cognitive testing in which the individual was ‘exposed’ (e.g if an individual reports a current smoker status for 10 of 30 visits, the cumulative exposure level is defined as 0.30). M=mean; SD=standard deviation; B=beta coefficient; CI=confidence interval; HAART=highly active antiretroviral therapy; ARV=antiretroviral. Bolded p-values denote those which are < 0.05 in multivariable-adjusted models.

**Supplemental Table 4. Characteristics of Virally Suppressed HIV-seropositive White/Other Women by Identified Baseline + Trajectory Clustering Group.**

| **Characteristic** | **Trajectory Group** | | **P-value** | |
| --- | --- | --- | --- | --- |
| Group [Color] in Supplemental Figure 2 | **High stable**  **[dark green]** | **Low stable**  **[light green]** | **Univariate** | **Multivariable Adjusted** |
| N | 29 | 65 |  |  |
| Average duration of time intervals between testing, mean (M) (Standard deviation [SD]) | 2.2 (0.5) | 2.1 (0.3) |  |  |
| Average duration of follow-up, years, M (SD) | 4.1 (2.0) | 4.4 (1.9) |  |  |
| *Declarative memory at baseline*** |  |  |  |  |
| Trial 1 learning, M (SD) | 11.76 (2.58) | 7.55 (2.29) | <0.0001 | **<0.0001** |
| Total learning, M (SD) | 11.88 (2.30) | 7.76 (1.87) | <0.0001 | **<0.0001** |
| Delayed recall, M (SD) | 12.29 (2.43) | 6.82 (2.59) | <0.0001 | **<0.0001** |
| Recognition, M (SD) | 11.32 (3.01) | 7.31 (3.01) | <0.0001 | **<0.0001** |
| *Rate of change in declarative memory per decade of age-years* |  |  |  |  |
| Trial 1 learning, Beta (B) (95% CI) | 1.43 (0.13, 2.73) | 1.24 (0.06, 2.41) | 0.01 | **0.002** |
| Total learning, B (95% CI) | 0.78 (-0.45, 2.02) | 0.71 (-0.41, 1.82) | 0.28 | 0.42 |
| Delayed recall, B (95% CI) | 1.35 (0.00, 2.71) | 1.20 (-0.03, 2.42) | 0.05 | 0.07 |
| Recognition, B (95% CI) | 0.45 (0.81. 1.70) | 0.40 (-0.73, 1.53) | 0.47 | 0.93 |
| *At initial cognitive test†* |  |  |  |  |
| Age, years, M (SD) | 43.0 (8.1) | 44.8 (5.5) | 0.29 | 0.17 |
| Hispanic ethnicity | 19 (29.7) | 17 (58.6) | 0.008 | 0.01 |
| Years of education, M (SD) | 14.0 (3.0) | 11.1 (3.3) | <0.0001 | **0.02** |
| WRAT-3 reading, M (SD) | 103.9 (12.0) | 90.6 (17.8) | <0.0001 | **0.02** |
| Annual household income (<$12k/year) | 16 (26.7) | 9 (13.8) | 0.60 | **0.05** |
| Employed | 37 (57.8) | 9 (31.0) | 0.02 | 0.66 |
| Depressed | 15 (23.4) | 13 (44.8) | 0.04 | 0.13 |
| Smokes | 12 (18.8) | 9 (31.0) | 0.20 | 0.83 |
| Heavy drinker | 1 (1.6) | 0 (0.0) | 0.39 | 0.09 |
| Marijuana use | 6 (9.4) | 4 (13.8) | 0.53 | 0.12 |
| Crack, Cocaine use | 0 (0.0) | 1 (3.5) | 0.12 | 0.15 |
| Heroin use | 0 (0.0) | 0 (0.0) | 1.00 | 0.15 |
| Body mass index, M (SD) | 27.5 (7.3) | 28.7 (6.8) | 0.46 | 0.21 |
| Hypertension | 23. (35.9) | 13 (44.8) | 0.42 | 0.34 |
| Diabetes | 13 (20.3) | 10 (34.5) | 0.15 | 0.88 |
| Hepatitis C RNA positive | 10 (15.6) | 7 (24.4) | 0.33 | 0.32 |
| Years of ARV | 12.1 (4.3) | 13.4 (3.8) | 0.20 | **0.05** |
| Years of HAART | 10.5 (4.2) | 10.6 (4.7) | 0.90 | 0.34 |
| CD4 count <200 | 1 (3.4) | 4 (6.2) | 0.56 | 0.22 |
| *% of WIHS visits prior to cognitive testing‡* |  |  |  |  |
| Annual household income (<$12k/year) | 26.4 (31.5) | 40.5 (37.3) | 0.06 | **0.05** |
| Employed | 57.3 (37.5) | 41.2 (39.7) | 0.06 | 0.52 |
| Depressed | 28.2 (32.3) | 44.1 (36.1) | 0.04 | **0.03** |
| Smokes | 21.8 (35.8) | 31.4 (35.9) | 0.23 | 0.83 |
| Heavy drinker | 2.1 (5.4) | 2.1 (6.9) | 1.00 | 0.75 |
| Marijuana use | 6.9 (16.0) | 14.3 (31.4) | 0.14 | 0.06 |
| Crack, Cocaine use | 2.0 (6.5) | 1.6 (6.3) | 0.81 | 0.31 |
| Heroin use | 1.6 (9.7) | 0.0 (0.0) | 0.38 | 0.06 |

*Adjusted for all factors included in the table. **Values presented are scaled to have a mean of 10 and a standard deviation of 3. †Calculated as the baseline (at initial cognitive visit) exposure level. ‡Calculated as a cumulative exposure level defined as the percentage of visits prior to cognitive testing in which the individual was ‘exposed’ (e.g if an individual reports a current smoker status for 10 of 30 visits, the cumulative exposure level is defined as 0.30). M=mean; SD=standard deviation; B=beta coefficient; CI=confidence interval; HAART=highly active antiretroviral therapy; ARV=antiretroviral. Bolded p-values denote those which are < 0.05 in multivariable-adjusted models.

**Supplemental Figure 1. Cluster Groups in HIV-seropositive (HIV+) Black/African American Virally Suppressed WIHS Women (n=166)**

**A.** Included declarative memory outcomes from the Hopkins Verbal Learning Test-Revised (HVLT-R): memory (delay free recall), learning (total learning), single trial learning (total words recalled on Trial 1), and recognition (number of words correctly identified on a yes/no recognition test). **B.** Included measures of motor function (time to completion of grooved pegboard test for dominant and non-dominant hands). Values plotted are linear fit of within group averages of scaled averaged across age and time.

**Supplemental Figure 2**. **Identified Cluster Groups in HIV-seropositive (HIV+) White/Other Virally Suppressed WIHS Women (n=94).**

**A.** Included declarative memory outcomes from the Hopkins Verbal Learning Test-Revised (HVLT-R): memory (delay free recall), learning (total learning), single trial learning (total words recalled on Trial 1), and recognition (number of words correctly identified on a yes/no recognition test). **B.** Included measures of motor function (time to completion of grooved pegboard test for dominant and non-dominant hands). Values plotted are linear fit of within group averages of scaled averaged across age and time.
